# Supplementary material for: Self-attention with temporal prior: can we learn more from the arrow of time?
Source: Front Artif Intell. 2024 Aug 6;7:1397298. doi: 10.3389/frai.2024.1397298 (PMC11333831; doi:10.3389/frai.2024.1397298)
Supplement: Supplementary file 1 [file Data_Sheet_1.pdf]

A. Appendix

A.1. Training and Hyperparameter Search

All of our implementations are in Tensorflow 1.13 and all experiments were done in NVIDIA Geforce RTX 2080Ti. For each model, we provide the following details on the explored hyperparameter search space. In order for more extensive hyperparameter tuning, we first performed smaller sets of test experiments to define the ranges of the values to search from. We then performed a grid search over all hyperparameter combinations from the found ranges, which are provided in the lists below. Because the eICU data set is much larger than other data sets, we defined a smaller search space due to the limitations in computational resources. The selected hyperparameters are presented in the Table 1.

The SAT-Transformer must learn kernel parameters in addition to the regular parameters of the Transformer. For ease of hyperparameter search, we optimized the Transformer part and the kernel part separately. The learning rate2 denotes the learning rate used to optimize kernel parameters which is given as multiplication factor to the learning rate used to optimize Transformer part of the network.

GRU-Simple

1) Physionet/MIMIC-III

learning rate $\in \{0.0001,\ 0.0002,\ 0.0005\}$
batch size $\in \{32,\ 64,\ 128\}$
hidden units $\in \{128,\ 256,\ 512\}$
dropout $\in \{0,\ 0.1,\ 0.2\}$
dropout recurrent $\in \{0,\ 0.1,\ 0.2\}$

2) eICU

learning rate $\in \{0.0001,\ 0.0002,\ 0.0005\}$
batch size $\in \{32,\ 64\}$
hidden units $\in \{256,\ 512\}$
dropout $\in \{0,\ 0.1\}$
dropout recurrent $\in \{0,\ 0.1\}$

GRU-D

1) Physionet/MIMIC-III

learning rate $\in \{0.0001,\ 0.0002,\ 0.0005\}$
batch size $\in \{32,\ 64,\ 128\}$
hidden units $\in \{128,\ 256,\ 512\}$
dropout $\in \{0,\ 0.1,\ 0.2\}$
dropout recurrent $\in \{0,\ 0.1,\ 0.2\}$

2) eICU

learning rate $\in \{0.0001,\ 0.0002,\ 0.0005\}$
batch size $\in \{32,\ 64\}$
hidden units $\in \{256,\ 512\}$
dropout $\in \{0,\ 0.1\}$
dropout recurrent $\in \{0,\ 0.1\}$

Interpolation Networks

1) Physionet/MIMIC-III

learning rate $\in \{0.0002,\ 0.0005\}$
batch size $\in \{16,\ 32,\ 64\}$
hidden units $\in \{128,\ 256,\ 512\}$
dropout $\in \{0,\ 0.1,\ 0.2\}$
dropout recurrent $\in \{0,\ 0.1\}$
reconstruction fraction $\in \{0.1,\ 0.2,\ 0.5\}$

2) eICU

learning rate $\in \{0.0002,\ 0.0005\}$
batch size $\in \{32,\ 64\}$
hidden units $\in \{128,\ 256\}$
dropout $\in \{0.1,\ 0.2\}$
dropout recurrent $\in \{0.0,\ 0.1\}$
reconstruction fraction $\in \{0.2,\ 0.5\}$

Transformer

1) Physionet/MIMIC-III

learning rate $\in \{0.0002,\ 0.0005,\ 0,001\}$
batch size $\in \{32,\ 64\}$
num of layers $\in \{2,\ 3,\ 4\}$
num of heads $\in \{2,\ 4,\ 8\}$
hidden units $\in \{256,\ 512\}$
dropout $\in \{0,\ 0.1,\ 0.2\}$

2) eICU

learning rate $\in \{0.0005,\ 0.001\}$
batch size $\in \{32,\ 64\}$
num of layers $\in \{3\}$
num of heads $\in \{2,\ 4,\ 8\}$
hidden units $\in \{256,\ 512\}$
dropout $\in \{0.1,\ 0.2\}$

SeFT

1) Physionet/MIMIC-III

learning rate $\in \{0.0005,\ 0.001,\ 0.002\}$
batch size $\in \{128,\ 256\}$
ϕ (layers) $\in \{2,\ 3,\ 4\}$
ϕ (width) $\in \{128,\ 256\}$
ρ (layers) $\in \{2,\ 3,\ 4\}$
ρ (width) $\in \{128,\ 256,\ 512\}$
ψ (layer) $\in \{2\}$
ψ (width) $\in \{64\}$
dot prod dim $\in \{128\}$
latent width: same as ϕ width
max time scale $\in \{1000\}$
pos dims $\in \{8\}$
dropout: randomly selected from $\{0.1,\ 0.2,\ 0.3,\ 0.4,\ 0.5\}$

2) eICU

learning rate $\in \{0.0005,\ 0.002\}$
batch size $\in \{128,\ 256\}$
ϕ (layers) $\in \{2,\ 4\}$
ϕ (width) $\in \{128,\ 256\}$
ρ (layers) $\in \{2,\ 3\}$
ρ (width) $\in \{256,\ 512\}$
ψ (layer) $\in \{2\}$
ψ (width) $\in \{64\}$
dot prod dim $\in \{128\}$
latent width: same as ϕ width
max time scale $\in \{1000\}$
pos dims $\in \{8\}$
dropout: randomly selected from$\{0.1,\ 0.2,\ 0.3\}$

SAT-Transformer

1) Physionet/MIMIC-III

learning rate $\in \{0, 0002,\ 0.0005,\ 0.001\}$
batch size $\in \{32,\ 64\}$
num of layers $\in \{2,\ 3,\ 4\}$
num of heads $\in \{2,\ 4,\ 8\}$
hidden units $\in \{256,\ 512\}$
dropout $\in \{0,\ 0.1,\ 0.2\}$
learning rate2 $\in \{\text{lrx20},\ \text{lrx50},\ \text{lrx100}\}$

2) eICU

learning rate $\in \{0.0005,\ 0.001\}$
batch size $\in \{32,\ 64\}$
num of layers $\in \{3\}$
num of heads $\in \{2,\ 4,\ 8\}$
hidden units $\in \{256,\ 512\}$
dropout $\in \{0.1,\ 0.2\}$
learning rate2 $\in \{\text{lrx20},\ \text{lrx50}\}$

A.2. Baseline Comparison

In this section, we compare the performances of the baseline models to other values in literature as shown in Table 2. The works done by (Horn et al., 2020; Che et al., 2018; Shukla & Marlin, 2019a), to our knowledge, show reliable performances of the same baseline networks. The evaluations done on PhysioNet or eICU data sets differ slightly from paper to paper. Due to this reason, the Table 2 only shows performances evaluated on MIMIC-III data set. We have tuned the hyperparameters of the baseline models to achieve at least the performances shown in these works, if not better, for the MIMIC-III data set, putting the same effort into other data sets as well.

	PhysioNet	MIMIC-III	eICU-HF	eICU-RF	eICU-KF
GRU-Simple	learning rate: 0.0002<br>batch size: 32<br>hidden units: 512<br>dropout: 0.2<br>dropout recurrent: 0.0	learning rate: 0.0002<br>batch size: 32<br>hidden units: 256<br>dropout: 0.1<br>dropout recurrent: 0.0	learning rate: 0.0001<br>batch size: 32<br>hidden units: 512<br>dropout: 0.1<br>dropout recurrent: 0.1	learning rate: 0.0001<br>batch size: 32<br>hidden units: 256<br>dropout: 0.1<br>dropout recurrent: 0.1	learning rate: 0.0005<br>batch size: 64<br>hidden units: 256<br>dropout: 0.1<br>dropout recurrent: 0.1
GRU-D	learning rate: 0.0002<br>batch size: 32<br>hidden units: 512<br>dropout: 0.2<br>dropout recurrent: 0.2	learning rate: 0.0002<br>batch size: 32<br>hidden units: 128<br>dropout: 0.2<br>dropout recurrent: 0.0	learning rate: 0.0005<br>batch size: 64<br>hidden units: 512<br>dropout: 0.1<br>dropout recurrent: 0.1	learning rate: 0.0005<br>batch size: 32<br>hidden units: 512<br>dropout: 0.1<br>dropout recurrent: 0.1	learning rate: 0.0005<br>batch size: 64<br>hidden units: 512<br>dropout: 0.1<br>dropout recurrent: 0.1
IP-Nets	learning rate: 0.0002<br>batch size: 16<br>hidden units: 128<br>dropout: 0.1<br>dropout recurrent: 0.0<br>reconstruction fraction: 0.1	learning rate: 0.0005<br>batch size: 32<br>hidden units: 128<br>dropout: 0.2<br>dropout recurrent: 0.0<br>reconstruction fraction: 0.5	learning rate: 0.0002<br>batch size: 64<br>hidden units: 256<br>dropout: 0.1<br>dropout recurrent: 0.0<br>reconstruction fraction: 0.2	learning rate: 0.0005<br>batch size: 32<br>hidden units: 128<br>dropout: 0.1<br>dropout recurrent: 0.0<br>reconstruction fraction: 0.5	learning rate: 0.0005<br>batch size: 64<br>hidden units: 256<br>dropout: 0.1<br>dropout recurrent: 0.0<br>reconstruction fraction: 0.2
Transformer	learning rate: 0.0002<br>batch size: 32<br>num of layers: 3<br>num of heads: 4<br>hidden units: 512<br>dropout: 0.2	learning rate: 0.0002<br>batch size: 64<br>num of layers: 3<br>num of heads: 8<br>hidden units: 512<br>dropout: 0.2	learning rate: 0.0005<br>batch size: 64<br>num of layers: 3<br>num of heads: 4<br>hidden units: 512<br>dropout: 0.2	learning rate: 0.001<br>batch size: 64<br>num of layers: 3<br>num of heads: 2<br>hidden units: 256<br>dropout: 0.1	learning rate: 0.0005<br>batch size: 32<br>num of layers: 3<br>num of heads: 8<br>hidden units: 512<br>dropout: 0.2
SeFT	learning rate: 0.001<br>batch size: 256<br>ϕ layers: 4<br>ϕ width: 128<br>ϕ dropout: 0.2<br>ρ layers: 2<br>ρ width: 512<br>ρ dropout: 0.1<br>ψ layer: 2<br>ψ width: 64<br>dot prod dim: 128<br>attention dropout: 0.2<br>latent width: 128<br>max time scale: 1000<br>pos dims: 8	learning rate: 0.0005<br>batch size: 128<br>ϕ layers: 2<br>ϕ width: 256<br>ϕ dropout: 0.2<br>ρ layers: 3<br>ρ width: 512<br>ρ dropout: 0.5<br>ψ layer: 2<br>ψ width: 64<br>dot prod dim: 128<br>attention dropout: 0.1<br>latent width: 256<br>max time scale: 1000<br>pos dims: 8	learning rate: 0.002<br>batch size: 256<br>ϕ layers: 4<br>ϕ width: 128<br>ϕ dropout: 0.1<br>ρ layers: 2<br>ρ width: 512<br>ρ dropout: 0.3<br>ψ layer: 2<br>ψ width: 64<br>dot prod dim: 128<br>attention dropout: 0.1<br>latent width: 128<br>max time scale: 1000<br>pos dims: 8	learning rate: 0.002<br>batch size: 256<br>ϕ layers: 4<br>ϕ width: 128<br>ϕ dropout: 0.1<br>ρ layers: 2<br>ρ width: 512<br>ρ dropout: 0.3<br>ψ layer: 2<br>ψ width: 64<br>dot prod dim: 128<br>attention dropout: 0.1<br>latent width: 128<br>max time scale: 1000<br>pos dims: 8	learning rate: 0.0005<br>batch size: 256<br>ϕ layers: 4<br>ϕ width: 128<br>ϕ dropout: 0.1<br>ρ layers: 2<br>ρ width: 512<br>ρ dropout: 0.1<br>ψ layer: 2<br>ψ width: 64<br>dot prod dim: 128<br>attention dropout: 0.2<br>latent width: 128<br>max time scale: 1000<br>pos dims: 8
SAT-Transformer	learning rate: 0.0002<br>batch size: 32<br>num of layers: 3<br>num of heads: 8<br>hidden units: 256<br>dropout: 0.1<br>learning rate2: x100	learning rate: 0.001<br>batch size: 64<br>num of layers: 2<br>num of heads: 8<br>hidden units: 256<br>dropout: 0.1<br>learning rate2: x20	learning rate: 0.0005<br>batch size: 32<br>num of layers: 3<br>num of heads: 4<br>hidden units: 512<br>dropout: 0.1<br>learning rate2: x20	learning rate: 0.0005<br>batch size: 32<br>num of layers: 3<br>num of heads: 8<br>hidden units: 512<br>dropout: 0.2<br>learning rate2: x50	learning rate: 0.0005<br>batch size: 32<br>num of layers: 3<br>num of heads: 4<br>hidden units: 512<br>dropout: 0.2<br>learning rate2: x20

Table A. Selected hyperparameters for each model for each task

AUROC/AUPR	Che et al.	Shukla et al.	Horn et al.	Ours
GRU-Simple	83.8 ± 0.8 / –	– / –	82.8 ± 0.0 / 43.6 ± 0.4	**85.4 ± 0.4 / 51.6 ± 0.6**
GRU-D	85.3 ± 0.3 / –	– / –	85.7 ± 0.2 / 52.0 ± 0.8	**86.1 ± 0.7 / 52.8 ± 0.5**
IP-Nets	– / –	**86.1 / 53.7**	83.2 ± 0.5 / 48.3 ± 0.4	85.4 ± 0.1 / 51.8 ± 0.9
Transformer	– / –	– / –	82.1 ± 0.3 / 42.6 ± 1.0	**84.8 ± 0.2 / 49.7 ± 0.3**
SeFT	– / –	– / –	83.9 ± 0.4 / 46.3 ± 0.5	**85.1 ± 0.3 / 46.2 ± 0.1**

Table B. Performances of the baseline models on MIMIC-III
